# Mechanical Properties and Microstructural Features of Biomass Fly Ash-Modified Self-Compacting Coal Gangue-Filled Backfill

**DOI:** 10.3390/ma16072789

**Published:** 2023-03-30

**Authors:** Guang Han, Zhifa Qin, Shenghao Zuo

**Affiliations:** 1 College of Safety Science and Engineering, Liaoning Technical University, Fuxin 123000, China; 2 School of Civil Engineering, Liaoning Technical University, Fuxin 123000, China; 3 School of Civil Engineering, Central South University, Changsha 410075, China

**Keywords:** biomass fly ash, cemented backfill, mechanical property, digital image correlation, microstructure

## Abstract

To achieve sustainable utilization of a large amount of mine solid waste, this study investigated the performance of self-compacting coal gangue-filled backfill (SCFB) containing biomass fly ash (BFA) generated from biomass power plants as a supplementary cementitious material (SCM). The correlations between the physical structure and compressive strength of SCFB samples were obtained by ultrasonic pulse velocity (UPV). The failure process of the SCFB samples was monitored by the digital image correlation (DIC) technique, and the stress–strain relationship and failure pattern were also analyzed. The micro-morphological structure and hydration products of SCFB samples were evaluated by X-ray diffraction (XRD), scanning electron microscopy (SEM), and backscattered electron imaging (SEM-BSE). The results show that the usage of 30~40% BFA in SCFB improves the physical structure and strength of the samples. The compressive strength and UPV value of SCFB samples with different water-to-cement (w/c) ratios showed a similar trend of increasing and then gradually decreasing as the proportion of ordinary Portland cement (OPC) replaced by BFA increased. BFA exhibits better reactivity and filling effect in SCFB samples with a high w/c ratio. The peak stress of SCFB samples gradually decreases, and resistance to deformation gradually weakens with the increase in w/c ratios, while the DIC results further verify the mechanical experimental results. Microstructural analysis revealed that reducing the w/c ratio and incorporating specific ratios of BFA can reduce the thickness of the interface transition zone (ITZ) and porosity. The results of the study will provide theoretical guidance for the modification, stability monitoring, and strengthening of SCFB.

## 1. Introduction

The continuous exploitation of mineral resources has significantly impacted the world’s economic development but also has produced negative consequences such as surface subsidence and the accumulation of large amounts of solid waste [1,2,3]. Solid waste management has gradually become a global issue because of the problems of land occupation, environmental pollution, and high management costs [4,5]. An important way to reduce the environmental impacts above is to utilize mine solid waste such as tailings sand (TS) and coal gangue (CG) to backfill the mined underground spaces, which not only solves the surface subsidence problem but also promotes sustainable mine development and green development of mineral resources [4,6]. Recently, many kinds of supplementary cementitious materials (SCMs) have been used to replace OPC as the main binder for cemented backfills, which brings advantages of reducing the environmental burden, optimizing the performances at both fresh and hardened states, and improving long-term durability, etc. [7,8,9,10].

Among various SCMs used in cemented backfills, existing reports claim that the partial replacement of OPC with biomass fly ash (BFA) facilitates zero-waste technology and sustainable development of solid waste [9,11,12]. In comparison with coal fly ash, the limited number of harmful elements in BFA also significantly reduces the adverse effects on human health and the natural environment [8,13]. Jaworska et al. [14] confirmed the feasibility of agricultural BFA as a mineral admixture in polymer-modified cement composites by examining its gradation and activity index and also revealed that the addition of BFA significantly enhances compressive strength. Thiedeitz et al. [7] used rice husk ash as SCM and investigated its contribution to mortar microstructure, strength, and durability properties; the enhanced strength development along with low water absorption and small capillary porosity indicated that rice husk ash is a suitable pozzolanic admixture. The reactivity of BFA is lower than OPC, and the mixture containing BFA usually increases the amount of water needed for maintaining the desired rheology of the fresh mixture [14]. It is known that the workability and mechanical strength of mixtures containing BFA depend on the dosage of BFA [14,15]. Fořt et al. [8] investigated the basic physical and mechanical properties of concretes modified with large amounts of BFA produced by wood combustion, and the results showed that BFA can replace cement binders up to 20 wt.% without any significant loss of mechanical properties, and even higher doses are still acceptable for low-performance applications. However, the chemical and phase composition diversity of BFA with various sources leads to different results being obtained for the properties of mixtures containing BFA.

The mechanical properties are considered to be key factors in the design of safe and robust cemented backfill structures [4,16]. The primary reason is that the physical structure of the cemented backfill must meet certain mechanical stability requirements to provide a safe underground working environment [4]. Moreover, the observation of structural defects is also essential to ensure the mechanical stability of the cemented backfill material, as defects are areas of stress concentration, and they constitute the cause of crack development and cause material damage [17,18,19]. Due to the heterogeneity and complexity of the SCFB structure in this study, the characterization of defects and cracks is significantly related to the determination of the test method [17]. The most significant methods for the detection and analysis of defects and cracks in SCFB composites include microscopic, ultrasonic methods based on acoustic analysis and digital image correlation (DIC) [17,20,21,22,23]. Studies have shown that the correlation between material physical structure and strength can be obtained indirectly through ultrasonic instruments [23,24,25]. A noncontact nondestructive optical measurement method known as digital image correlation (DIC) has been widely used in civil engineering research with the rapid development of image processing technology, which can accurately calculate the displacement and strain during loading [26,27,28]. Bakir et al. [29] used the DIC method to detect the deformation behavior of composite materials in bending tests, and the mechanical simulation results of DIC processed by the open-source 2D digital image correlation MATLAB software (NCORR) were consistent with the experimental test results. Moreover, DIC technology can effectively track the crack shape and expansion pattern of the test object in real-time, which overcomes the deficiencies of traditional inspection methods and obtains more complete information on the deformation response of the damage behavior of the cemented backfill [22,27].

This work investigated the feasibility of BFA for modifying SCFBs, which may provide a sustainable alternative to OPC. The effect of BFA produced from a biomass power plant on the strength of SCFBs and their mechanical response under loading was determined by studying the compressive strength and the microstructure of the SCFB samples. Therefore, the effect law of different replacement percentages of OPC by BFA on the compressive strength of SCFB samples with different water-to-cement (w/c) ratios and different curing ages was first investigated experimentally, and the relationship between the physical structure and strength of SCFB samples was characterized by ultrasonic pulse velocity (UPV). Secondly, mechanical tests combined with the digital image correlation (DIC) technique were used to quantitatively describe the failure process of SCFB samples under compressive loading, and the stress–strain behavior and failure patterns of SCFB samples were also analyzed. Finally, X-ray diffraction (XRD), scanning electron microscopy (SEM), and backscattered electron imaging (SEM-BSE) were used to comprehensively analyze the microstructural characteristics of SCFB samples, and the influence mechanism of BFA on the macroscopic properties of SCFB samples was also elucidated.

## 2. Materials and Methods

### 2.1. Materials

**Figure 1:**
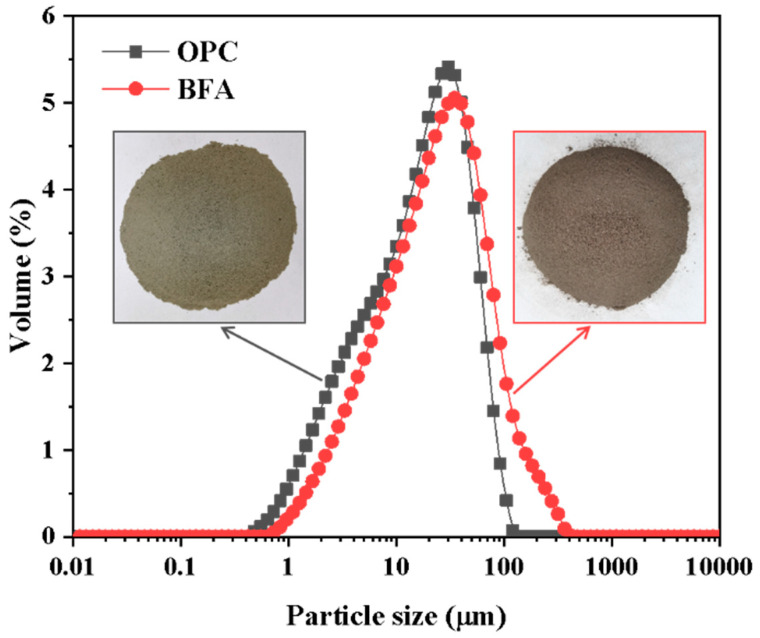
Particle size distributions and appearances of OPC and BFA.

In this study, P∙O 42.5 OPC was used, and BFA was obtained from a biomass power plant in Fuxin, Liaoning, China. The specific surface areas of OPC and BFA are 941 and 605 m^2^/kg, respectively. The chemical compositions of OPC and BFA are illustrated in Table 1, and the particle size distributions and appearances are presented in Figure 1. The TS and CG were from a local iron tailings dam and a coal mine, respectively. The apparent densities of TS and CG are 2.67 and 2.35 g/cm^3^, respectively. After the screening, TSs and CGs with particle sizes ranging from 0 to 2 mm and 16 to 25 mm were used, respectively. More details of TSs and CGs have been reported in our previous studies [2,6]. Tap water was used for all experiments in this study.

### 2.2. Mixture Proportion and Sample Preparation

The mixture proportions of SCFB samples are presented in Table 2. Four different dosages (i.e., 0%, 30%, 40%, and 50%, by mass of cement) of BFA were considered in the OPC replacement according to our published studies [2,6]. In this study, the solid mass fraction of 60% and w/c ratios of 0.8 and 1.0 were chosen to prepare the fresh TS pastes. The workability of fresh TS pastes was identified based on GB/T 2419-2005 and ASTM C 939-02 [6]. The flow diameters of the fresh TS pastes in this study were controlled in the range of 25 to 40 mm according to the minimum viscosity method, while the flow times were measured to be less than 15 s according to the flow cone method; in this way, no significant bleeding or separation were observed among the mixtures.

**Figure 2:**
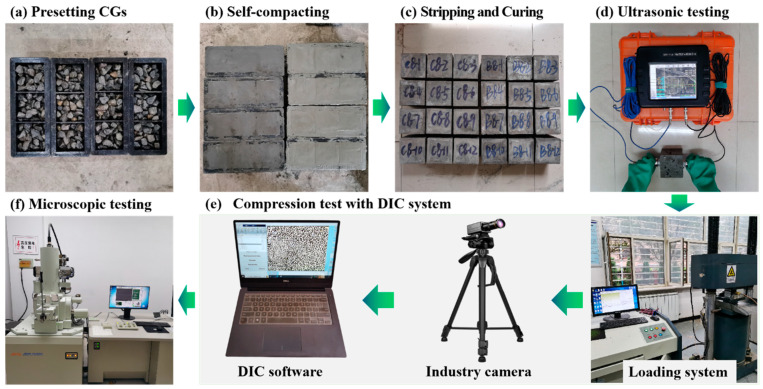
The preparation process and test methods on SCFB samples.

When preparing the SCFB samples, the powdered materials were dry mixed in an electric mixer, and then water was added. Afterward, it was mixed at low speed for 1 min, then at high speed for 3 min, and then at low speed for 1 min. Finally, the homogeneous fresh TS pastes were poured into a mold (100 × 100 × 100 mm^3^) pre-placed with CG particles, demolded after 48 h, and standard cured (20 ± 2 °C, 98% RH) to the design age. The preparation process and test methods on SCFB samples are shown in Figure 2.

### 2.3. Testing Methods

#### 2.3.1. Mechanical Property Tests

The uniaxial compressive strength of SCFB samples (100 × 100 × 100 mm^3^) was measured after standard curing to the specified age (i.e., 28 and 56 days) according to GB/T50081-2016. The compressive strength test was performed using the Hydraulic servo testing machine in stress control mode with a loading rate of 5 kN/s. Furthermore, the UPV was measured using the GTJ-U820 ultrasonic instrument according to CECS 21:2000.

#### 2.3.2. Behavior under Mechanical Loads Using DIC

To analyze the failure characteristics of SCFB samples under uniaxial compression loading, the surface failure characteristics of the samples were evaluated with the DIC monitoring system. In this study, the images were collected with a 3 million USB HD color industrial camera fitted with a CW05100-A industrial lens. The photographs were automatically stored in a computer folder specified by the MindVision measurement software, and the shooting frequency was set to 1 shot/s. To facilitate the measurement of deformation during the compressive test, randomly scattered spots were created on the surface of the SCFB samples by using a marker. Before starting the test, the camera was placed in a uniformly lighted area for photometric tuning until the system captured a photograph in which the scattered spots could be observed. The open-code Ncorr software developed by the Georgia Institute of Technology was used in this study to determine the deformation behavior of the SCFB samples by consistently comparing the position of the scattered spots before and after the test [29].

#### 2.3.3. Microstructural Analyses

The secondary electron images were collected using scanning electron microscopy (SEM, TESCAN MIRA LMS, Brno, Czech Republic) for microscopic min eral morphology. The ITZ between the TS paste and the CG particles was investigated by collecting backscattered electron images (SEM-BSE, TESCAN MIRA LMS, Brno, Czech Republic). The samples were also used for X-ray diffraction analysis (XRD, Rigaku SmartLab SE, Tokyo, Japan) according to the mixture ratios of SCFBs without the use of TS and CG. The sample was ground into a powder with a particle size of less than 0.075 mm and scanned from 5° to 60° at a speed of 10 °/min.

#### 2.3.4. Cost and Environmental Index

The cost and environmental impacts are essential factors for evaluating the feasibility of SCFB in engineering applications. The costs and CO_2_ emissions of raw materials are listed in Table 3. It should be noted that CG, TS, and BFA used in this study are considered solid wastes, and the costs and CO_2_ emissions of TS and BFA are derived from transporting them to the mined sites, with the transportation distance of 25 and 35 km for TS and BFA, respectively [6]. The calculation of strength efficiency, cost index (C_P_, USD/m^3^·MPa), and embodied CO_2_ index (CI, kg/m^3^·MPa) of SCFBs can be found in the Refs. [2,30].

## 3. Results and Discussion

### 3.1. Compressive Strength and UPV Results

**Figure 3:**
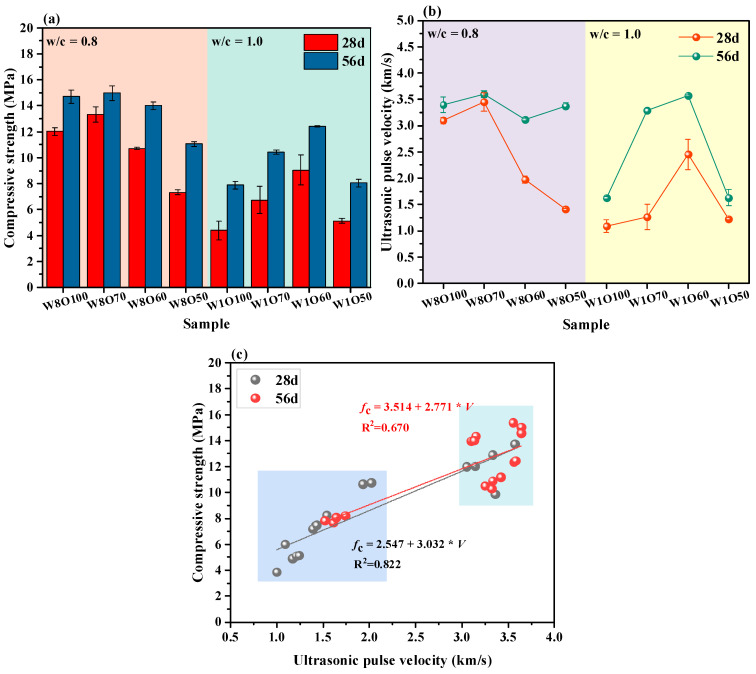
Compressive strength (**a**), UPV values (**b**) and the fitting relationship of compressive strength vs. UPV (**c**) of SCFB samples at 28 and 56 d.

The compressive strength of the SCFB samples at 28 and 56 d are shown in Figure 3a. As seen, the compressive strength of SCFB samples is mainly related to the w/c ratio, the curing time, and the replacement percentage of OPC by BFA. The compressive strength of SCFB samples decreases with the increase in w/c ratios, while an uptrend of the compressive strength is observed with the increase in curing age. Furthermore, the compressive strength of SCFB samples with different w/c ratios first increases and then gradually decrease as the replacement percentage of OPC by BFA increases. In general, the compressive strength of the SCFB samples with different w/c ratios reached the maximum value at replacement percentages of 30% and 40%.

As known, the UPV is a non-destructive test method for determining the homogeneity and physical structure monitoring of material structures [23,24]. The SCFB samples in this study were prepared by the self-compacting method. The difference in the workability of fresh TS pastes usually leads to the possibility of partial flaws in the microstructure of SCFB samples. Therefore, the UPV is used to characterize the microstructural integrity of the SCFB samples. The UPV of the SCFB samples at 28 and 56 d is shown in Figure 3b. It can be seen that the UPV values gradually increase with the increase in curing time. A similar trend to the compressive strength is that the UPV values generally tend to increase and then decrease with the increase in replacement percentages of BFA.

The correlation between compressive strength and UPV values is illustrated in Figure 3c. In general, a higher UPV value represents a denser structure of the SCFB samples, which results in better compressive strength. Jiang et al. [4] also identified that cemented backfills with relatively high porosity usually have less compressive strength. As seen in Figure 3c, a relatively high correlation coefficient between UPV values and compressive strength is observed at different curing ages. In the lower compressive strength range, the poor filling ability and the inhomogeneous structure of the SCFB samples result in lower UPV values. The UPV value increases nearly linearly with the compressive strength, which is consistent with the results reported in previous work [33]. The SCFB samples with higher compressive strength typically exhibit a denser structure with the increase in curing time. As a result, the UPV values then exhibit relatively high and stable values (i.e., in the range of 3.0 to 4.0 km/s), resulting in a relatively low linear correlation coefficient (i.e., R^2^ = 0.670). Therefore, the UPV values of the SCFB samples in this study verify and validate the results for compressive strength.

### 3.2. Cracking Development and Failure Modes Investigated by DIC

**Figure 4:**
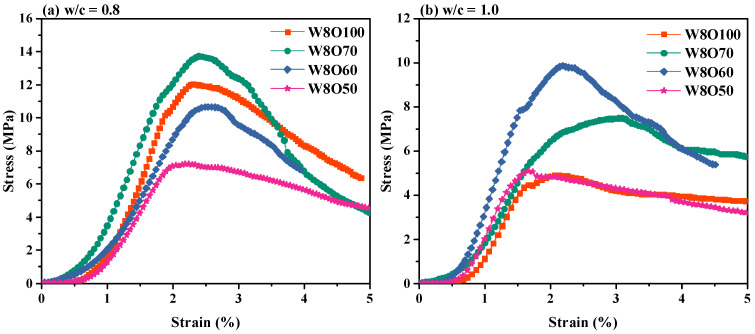
Stress–strain curves of SCFB samples at 28d.

The stress–strain curves of SCFB samples at 28d are presented in Figure 4. As seen, the peak strains exhibit a similar tendency to increase and then decrease with the increase in replacement ratios. At low w/c ratios (i.e., 0.8), the deformation modulus E50 (i.e., defined at 50% of peak stress) is 4.013, 5.067, 3.457, and 2.573 MPa with the increase in replacement ratios. The peak stress and deformation modulus E50 are the highest for SCFB samples with a 30% replacement ratio. Comparably, the deformation modulus E50 with the increase in replacement ratio is 1.981, 2.757, 4.139, and 2.377 MPa for high w/c ratios (i.e., 1.0). However, the SCFB samples with a replacement rate of 40% exhibited the highest peak stress and deformation modulus E50. Therefore, a reasonable replacement of OPC by BFA with different w/c ratios can increase the peak stress and deformation modulus E50, which leads to enhanced deformation resistance of SCFB samples.

**Figure 5:**
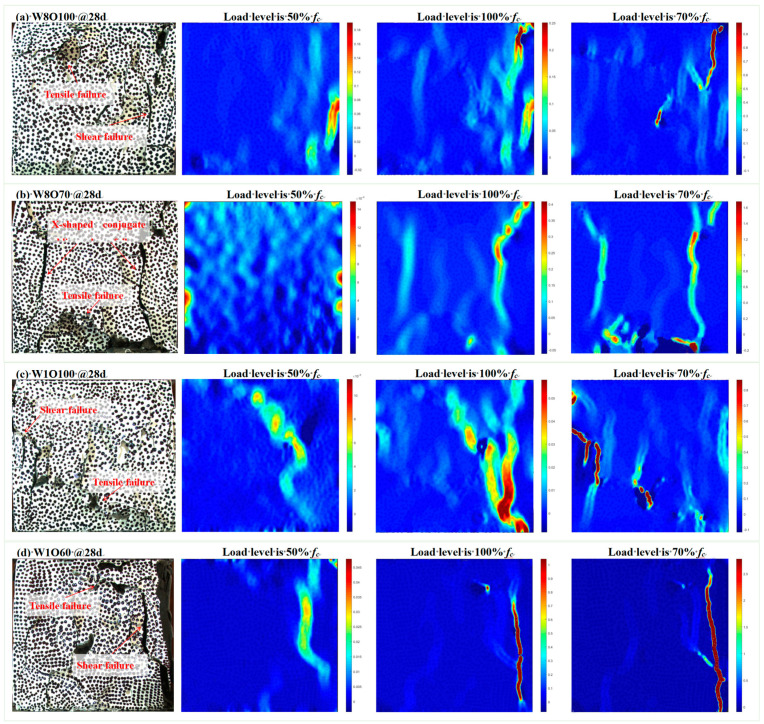
The failure process of SCFB samples was measured by DIC.

As known, DIC is an image correlation algorithm that can be used to evaluate the surface strain characteristics and crack development of materials [19,34]. The crack development of SFCB samples was monitored during uniaxial compressive testing in this study using the DIC technique, and the crack development at different load levels is shown in Figure 5. The target area used for crack monitoring in the DIC is 100 × 100 mm^2^, and the horizontal strain ε_xx_ cloud diagrams for different stress levels are calculated and presented. As seen, the horizontal strains ε_xx_ of the SCFB samples (i.e., W8O100, W8O70, W1O100, and W1O60) are significantly distinct at different stress levels. The peak stress of SCFB samples gradually decreases, and resistance to deformation becomes weaker with the increase in w/c ratios, which leads to the horizontal strain ε_xx_ of the SCFB samples gradually increasing at 50% and 100% stress levels. This may be due to the lower hardened TS paste strength and weaker ITZ (i.e., located between the TS pastes and CG particles) of the SCFB samples with high w/c ratios, which allows for more rapid failure by the formation of numerous microcracks during the failure process [18]. In addition, the horizontal strain ε_xx_ of W8O70 and W1O60 samples is greater than that of W8O100 and W1O100 samples, respectively, at 100% stress level and 70% post-peak stress level. This is due to the increase in peak stress and brittleness of SCFB samples and the large horizontal strain ε_xx_ caused by the penetration and expansion of macroscopic large cracks in SCFB samples. It should be noted that the strain cloud diagrams of crack development may be incomplete during the residual strength stage due to the rapid increase in relative displacement and varying degrees of peeling of the outer surface of the SCFB samples (in Figure 5).

As shown in Figure 5, the failure morphology of the SCFB samples is basically similar, i.e., the samples mainly exhibit shear failure and tensile failure. It should be noted that the failure behavior of the SCFB samples with different w/c ratios is different. When the strength is relatively low, both the strength of the TS paste and the bond strength between the TS paste and CG particles in the SCFB samples are also low, and the small cracks gradually penetrate and eventually form large cracks mainly in tensile failure during the compression process (see Figure 5c). On the contrary, the strength of the TS paste and the bond strength between TS paste and CG particles in the SCFB samples are both higher when the strength is relatively high, the outer layer of the sample peels off along the crack path during the compression process, and the main cracks on both sides of the sample penetrate to form a typical X-shaped conjugate oblique shear failure (see Figure 5b).

### 3.3. Microstructural Features

**Figure 6:**
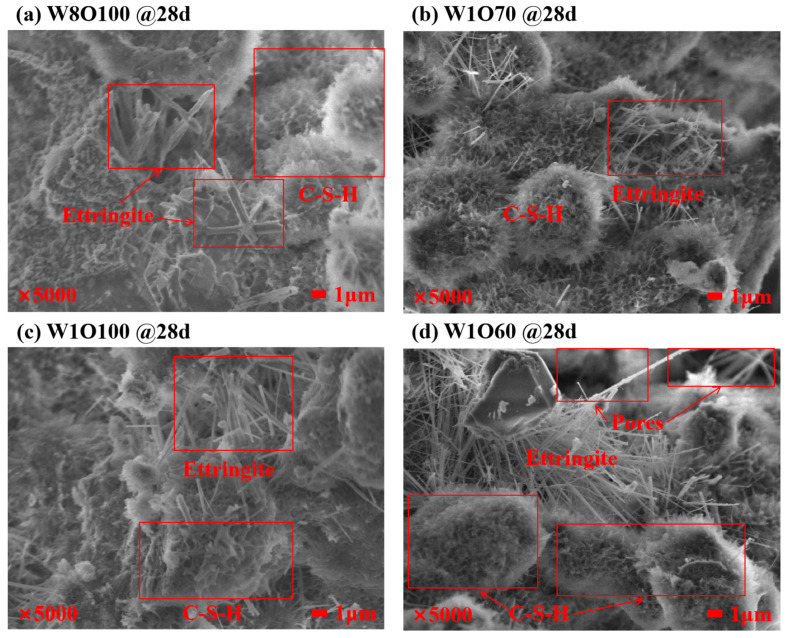
Microscopic features of SCFB samples with different w/c ratios and replacement ratios.

**Figure 7:**
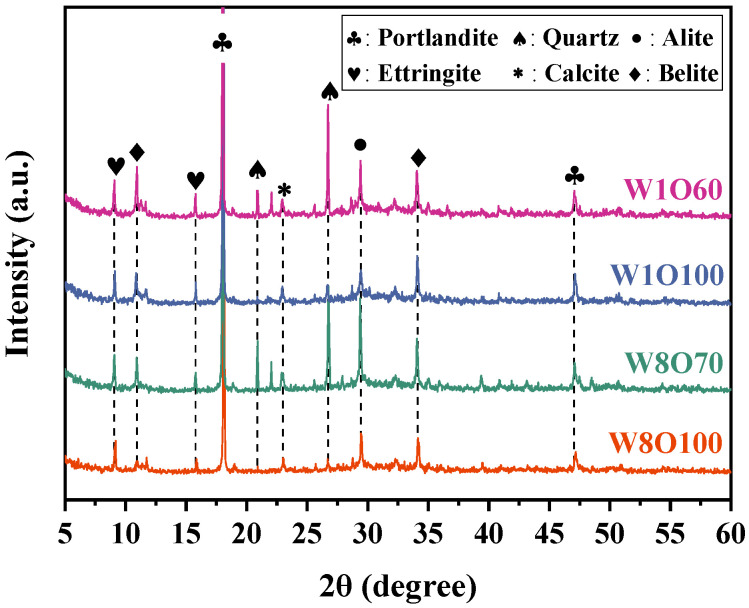
XRD pattern of SCFB samples at 28 d.

The microscopic morphological structures and hydration products of SCFB samples were evaluated by SEM. Figure 6 shows the microscopic features of the SCFB samples with different w/c ratios and replacement ratios at 28 d. As seen, the most remarkable hydration products at this stage are ettringite crystals with different lengths and diameters in the pores, and the dense and continuous coverage of C-S-H gels with different shapes is also observed on the grain surfaces. Moreover, the open pore number of SCFB samples with a w/c ratio of 1.0 significantly increases with the increase in replacement percentages (see Figure 6c,d). The XRD pattern of the SCFB samples at 28 d is presented in Figure 7. As seen, the main crystalline phases are portlandite, ettringite, quartz, and calcite, and some unhydrated cement clinkers such as allite and bilite can also be noted. Similar results have been reported in [6]. The diffraction peaks of quartz were significantly enhanced after the replacement of OPC by BFA, while the diffraction peaks of hydration products were not significantly affected.

**Figure 8:**
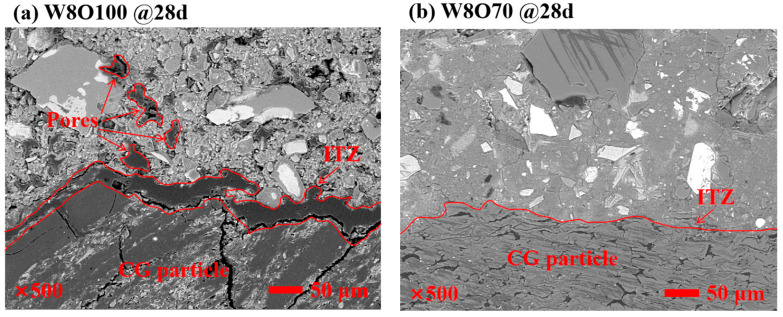

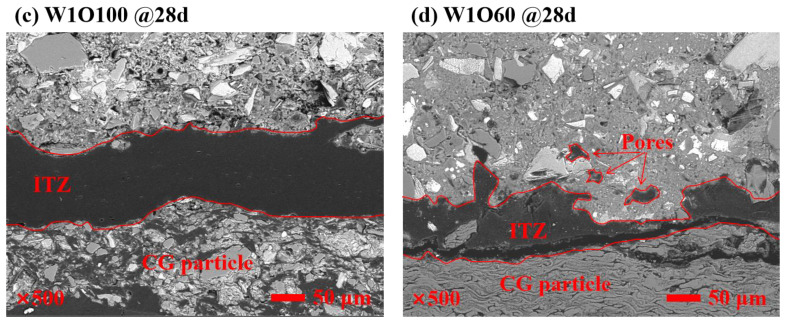
SEM-BSE image of SCFB samples at 28 d.

As known, the ITZ thickness within the multiphase composites exhibits a certain correlation with the macroscopic mechanical properties [21,35,36]. The SCFB in this study is a self-compacting multiphase composite material by casting the highly flowable fresh TS paste into the skeleton of pre-placed CG particles, whose mechanical properties mainly depend on the strength of TS pastes and the bonding interface strength between TS paste and CG particles. The SEM-BSE images of SCFB samples with different w/c ratios at 28 d are illustrated in Figure 8. It can be found that the ITZ thickness between TS paste and CG particles increases significantly with the increase in the w/c ratio. The ITZ thickness between TS paste and CG particles is significantly decreased at the optimal replacement ratio of OPC by BFA in SCFB samples with different w/c ratios (see Figure 8b,d). Moreover, it can be found that the existence of BFA decreases the porosity near ITZ of SCFB samples with a low w/c ratio; on the contrary, the porosity near ITZ of SCFB samples with a high w/c ratio increases.

Since the preparation of SCFB in this study was not involved in the stirring process, the bonding interface between the TS paste and the CG particles was formed by the TS paste filling the voids between the CG particles and coming into contact with them. As a result, the interface formed is relatively weak. The important role of CG particles in SCFB samples as a skeleton inhibits the direction of possible crack development at the interfaces or in the TS pastes [37]. Moreover, dry CG particles can absorb water from TS paste, which reduces the w/c ratio at the bonding interface. De la Varga et al. [38] indicated that water transport can generate internal stresses at the interface, making it become a weak site. Therefore, the final strength of SCFB samples when stressed is determined by the worst strength of the bonding interface between TS paste and CG particles, TS paste matrix, and CG particles. The failure occurs usually at the bonding interface between the TS paste and CG particles or in the TS paste. However, some CG particles can also break under stress concentration, which is because part of the force may be transferred directly between CG particles when being compressed [37].

In general, the potential mechanism of the w/c ratio for the cementitious material is mainly to increase the free water content and pore content [20], while the role of BFA in the cementitious material mainly includes the potential pozzolanic activity effect and the filling effect [13,39]. With the increase in the w/c ratio, the rate of OPC replacement by BFA increased by an additional 10% at the same strength as the comparison group (See Figure 3a). This indicates that BFA exhibits better reactivity and filling effect in SCFB samples with a high w/c ratio. Previous studies have shown that the w/c ratio has a more significant hydration promotion effect on low reactive admixtures [40]. The replacement of OPC by BFA not only reduces the OPC content per unit volume but also the filling effect of BFA particles by providing more hydration space [6,7]. The high w/c ratio provides more available water and space for the reaction of the cement clinker, which not only promotes the hydration of the cement but also facilitates the dissolution of the BFA. However, this also cannot compensate for the increased porosity and ITZ thickness brought by the high w/c ratio. Therefore, this further elaborates on the underlying factors causing the differences in the macroscopic mechanical properties of the SCFB samples.

### 3.4. Evaluations of Economic and Environmental Impacts

**Figure 9:**
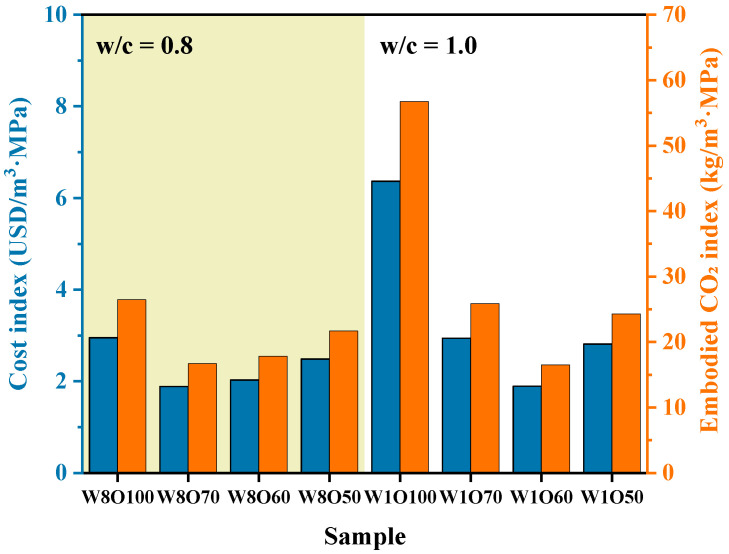
The cost and environmental impact assessment of the SCFBs (blue: cost index; orange: embodied CO_2_ index).

The cost Cp and embodied environmental impact CI of SCFBs with different w/c ratios are presented in Figure 9. It can be seen that the usage of 30~40% BFA in SCFB can significantly reduce the Cp and CI values. As the replacement rate of BFA increases, the Cp and CI values of SCFB show a similar trend of decreasing and then increasing significantly. Although BFA has a lower cost, the SCFB samples mixed with the large volume of BFA have relatively low compressive strength at 28 d. Moreover, the Cp and CI values of SCFB increase significantly with the increase in the w/c ratio. The dosage of OPC per unit volume is reduced in this case, but the Cp and CI values are also closely related to the compressive strength [30]. Therefore, the partial replacement of OPC by BFA can achieve good economic and environmental benefits.

## 4. Conclusions

The mechanical properties of BFA-modified SCFBs with different w/c ratios and different replacement percentages of BFA were investigated, the stress–strain relations and failure behavior of SCFB samples were analyzed by the DIC technique, and the effect of BFA on the microstructure of SCFB samples was evaluated by XRD, SEM, and SEM-BSE tests. Based on the experimental results and discussion, the conclusions can be summarized as follows:

1. The compressive strength of SCFB can be increased by incorporating BFA at a 30% replacement rate at a low w/c ratio, and the replacement rate of BFA incorporation can be up to 40% at a high w/c ratio. BFA exhibits better reactivity and filling effect in SCFB samples with a high w/c ratio.
2. The peak strain and deformation modulus E50 show a similar trend of increasing and then decreasing with the increase in replacement ratio. The relatively high-strength SCFB samples formed a typical x-type conjugate oblique shear failure, while the relatively low-strength SCFB samples were damaged mainly in tensile failure and secondarily in shear failure.
3. Decreasing the w/c ratio can reduce the microscopic porosity and at the same time can reduce the thickness of ITZ, which is beneficial to improving the physical structure and strength of SCFB samples. The ITZ thickness can be reduced when OPC is partially and reasonably replaced by BFA, and the microscopic porosity of SCFB samples can be reduced at a low w/c ratio.
4. Considering the application of SCFBs in engineering, using 30~40% BFA to replace OPC is an ideal choice because it has lower costs and lower carbon emissions.

## Figures and Tables

**Table 1:** Chemical compositions of OPC and BFA [6].

Chemicals (wt/%)	CaO	SiO_2_	Al_2_O_3_	Fe_2_O_3_	MgO	SO_3_	Other Oxides	Loss on Ignition
OPC	62.80	20.60	4.13	2.99	1.93	2.56	3.93	1.06
BFA	4.19	58.76	11.66	4.5	3.24	1.28	9.48	6.89

**Table 2:** Mixture proportions of SCFBs.

Sample	w/c Ratios	Water (g)	OPC (g)	BFA (g)	TS (g)
W8O100	0.8	1000.0	1250.0	0	250.0
W8O70	0.8	1000.0	875.0	375.0	250.0
W8O60	0.8	1000.0	750.0	500.0	250.0
W8O50	0.8	1000.0	625.0	625.0	250.0
W1O100	1.0	1000.0	1000.0	0	500.0
W1O70	1.0	1000.0	700.0	300.0	500.0
W1O60	1.0	1000.0	600.0	400.0	500.0
W1O50	1.0	1000.0	500.0	500.0	500.0

Note: W stands for the w/c ratios; O stands for the OPC content in the binder. W8O70 means that the w/c ratio is 0.8, and the binder is composed of 70% OPC and 30% BFA.

**Table 3:** Costs and CO_2_ emissions of raw materials.

Raw Materials	Cost per 1000 kg (USD)	CO_2_ Emission (kg/kg)	References
CG	N/A	N/A	-
TS	1.42	0.62 × 10^−4^	[31]
OPC	80.00	0.72	[32]
BFA	1.99	0.87 × 10^−4^	[6]

## Data Availability

Date can be obtained from corresponding authors upon reasonable request.
